# Greenness assessment of two chromatographic methods developed for the determination of Mupirocin in two binary mixtures along with its impurity

**DOI:** 10.1186/s13065-023-01055-5

**Published:** 2023-10-27

**Authors:** Maha M. Galal, Shaaban A. Abdullah, Ola Y. Mohamed, Azza A. Moustafa

**Affiliations:** 1 Analytical Chemistry Department, Faculty of Pharmacy, Cairo University, Kasr El-Aini street, Cairo, 11562 Egypt; 2 Egyptian Drug Authority-Central Administration for Drug Control (EDA-CADC), Cairo, Egypt

**Keywords:** Mupirocin, Pseudomonic acid-D, HPLC, HPTLC, Greenness assessment

## Abstract

Two simple, accurate and precise chromatographic methods have been developed and validated for estimating Mupirocin (MUP) in two binary mixtures. Mixture (1); with Fluticasone propionate (FLU) together with two of their impurities, namely; Pseudomonic acid-D (Pseud-D) and Fluticasone impurity C (FIC). Mixture (2); with Mometasone furoate (MF) along with Pseud-D impurity. High performance thin layer chromatography (HPTLC-densitometry) and high performance liquid chromatography (RP-HPLC) were the two proposed methods. In the HPTLC method, good separation of both mixtures was achieved by using HPTLC plates pre-coated with silica gel 60 F_254_ as stationary phase and the mobile phase consisted of toluene: chloroform: ethanol at a ratio of (5: 4: 2, by volume). The detection was carried out at 220 nm for MUP and 254 nm for FLU, MF, Pseud-D and FIC. In the HPLC method, chromatographic separation was carried out using Agilent Eclipse XDB (250 mm×4.6 mm, 5 μm) C18 column. For mixture (1), a mobile phase of methanol: sodium di-hydrogen phosphate (pH 3.0) was applied in stepwise gradient elution starting at ratios of (50: 50, v/v) and then switching to (80: 20, v/v) after 7 min at a flow rate of 1 mL.min^− 1^. Detection was performed using diode array detector at 220 nm for MUP and Pseud-D and 240 nm for FLU and FIC. For mixture (2), the same mobile phase was used, but in isocratic elution in the ratio (80: 20, v/v) at flow rate of 1 mL.min^− 1^ and detection at 220 nm for MUP and Pseud-D and 248 nm for MF. The two methods successfully separated the cited drugs and were used to determine the drugs in pure form as well as pharmaceutical dosage forms. Validation was done as per International Council on Harmonization guidelines. Furthermore, the greenness of the proposed methods compared to the reported method, was evaluated as per the National Environmental Method Index, analytical Eco scale, Green Analytical Procedure Index and Analytical Greenness metric approaches.

**Supplementary Information:**

The online version contains supplementary material available at 10.1186/s13065-023-01055-5.

## Introduction

Green chemistry was designed to save the environment by reducing the usage of toxic solvents. Green analytical chemistry (GAC) concepts are used to reduce environmental pollution and improve the health of analysts [1]. Several researches are reported in the literature for the determination of active pharmaceutical ingredients (APIs) either alone or in combination with their impurities using green analytical approaches [2–4].

Impurities are well-defined in the pharmaceutical industry as unwanted substances that remain with APIs or develop during the formulation or aging of both APIs and formulations [5].

Different regulatory authorities, such as the Food and Drug Administration (FDA) and the International Council on Harmonization (ICH), highlight on the purity requirements and the identification of impurities in APIs, revealing the need and scope of drug impurity profiling in pharmaceutical research. As a result, and in accordance with ICH guidelines, it is critical to develop an analytical method capable of identifying and determining impurities [6]. Furthermore, the method must be capable of resolving all impurities from the parent drug as well as from each other [7].

Mupirocin (MUP) is an official drug in the British pharmacopoeia (BP) [8] and the United States pharmacopeia (USP) [9]. It is chemically known as: 9-[(E)-4-[(2 S, 3R, 4R, 5 S) 3, 4-dihydroxy-5-[[(2 S, 3 S)-3-[(2 S, 3 S) hydroxylbutan-2-yl] oxiran-2-yl] methyl] oxan-2-yl] − 3methylbut-2-enoyl] oxynonanoic acid. MUP, bactroban or Pseudomonic acid is a topical antibacterial agent initially produced by fermentation using the organism *Pseudomonas fluorescens*. MUP is used in the treatment of impetigo and traumatic skin lesions resulting from secondary skin infections caused by susceptible aerobic gram-positive cocci, like *Staphylococcus aureus, Staphylococcus epidermidis*, and other beta-hemolytic streptococci *Streptococcus pyogenes* [10].

USP states 9 official impurities for MUP, MUP impurities 1, 2, 3, 4 and 5 and Pseudomonic acids B, C, D and F [9]. By surveying the literature, no published methods were found for determining MUP together with any of its impurities.

Fluticasone propionate (FLU) is an approved medication in the BP [8] and the USP [9]. It is chemically known as (6α,11β,16α,17α)-6,9-Difluoro-11-hydroxy-16-methyl-3-oxo-17-(1-oxopropoxy)androsta-1,4-diene-17-carbothioate, 17-propanoate. It is a moderately potent synthetic corticosteroid that is used topically to treat dermatoses and psoriasis, as well as intranasally to handle allergic and non-allergic rhinitis symptoms [11, 12]. BP also states 5 official impurities for FLU; fluticasone propionate impurities A, B, C, D, E. Due to the structural similarity between FLU and FIC, FIC can be used as a secondary standard of FLU [8].

There have been two reported chromatographic techniques for determining FLU in the presence of its impurities [13, 14].

Mometasone furoate (MF) is a USP - approved drug [9]. It is chemically known as 9, 21 – dichloro-11b, 17 – dihydroxy-16a-methyl-pregnane-1, 4, lodiene – 3, 20 – Dione 17 – (2 – furoate ester). It is a topical corticosteroid which has anti-pruritic, anti-inflammatory and vasoconstrictive properties. It is specified for a number of disorders such as allergic reactions, eczema and psoriasis [15].

MUP is formulated in the form of topical ointment either with FLU (Flutibact®) or with MF (Metos-M®). Flutibact® is used in the treatment of atopic dermatitis [12] whereas Metos-M® is used in the treatment of dermatitis of the scalp, impetigo and other conditions [16].

Three chromatographic HPLC methods have been reported for the simultaneous determination of MUP and FLU [12, 17, 18]. Furthermore, only two methods were found for the determination of MUP and MF, one of them was HPLC [16] and the second was spectrophotometric one [19]. Nonetheless, none of these methods took into account the concurrent determination of the cited drugs in conjunction with their impurities.

**Fig. 1:**
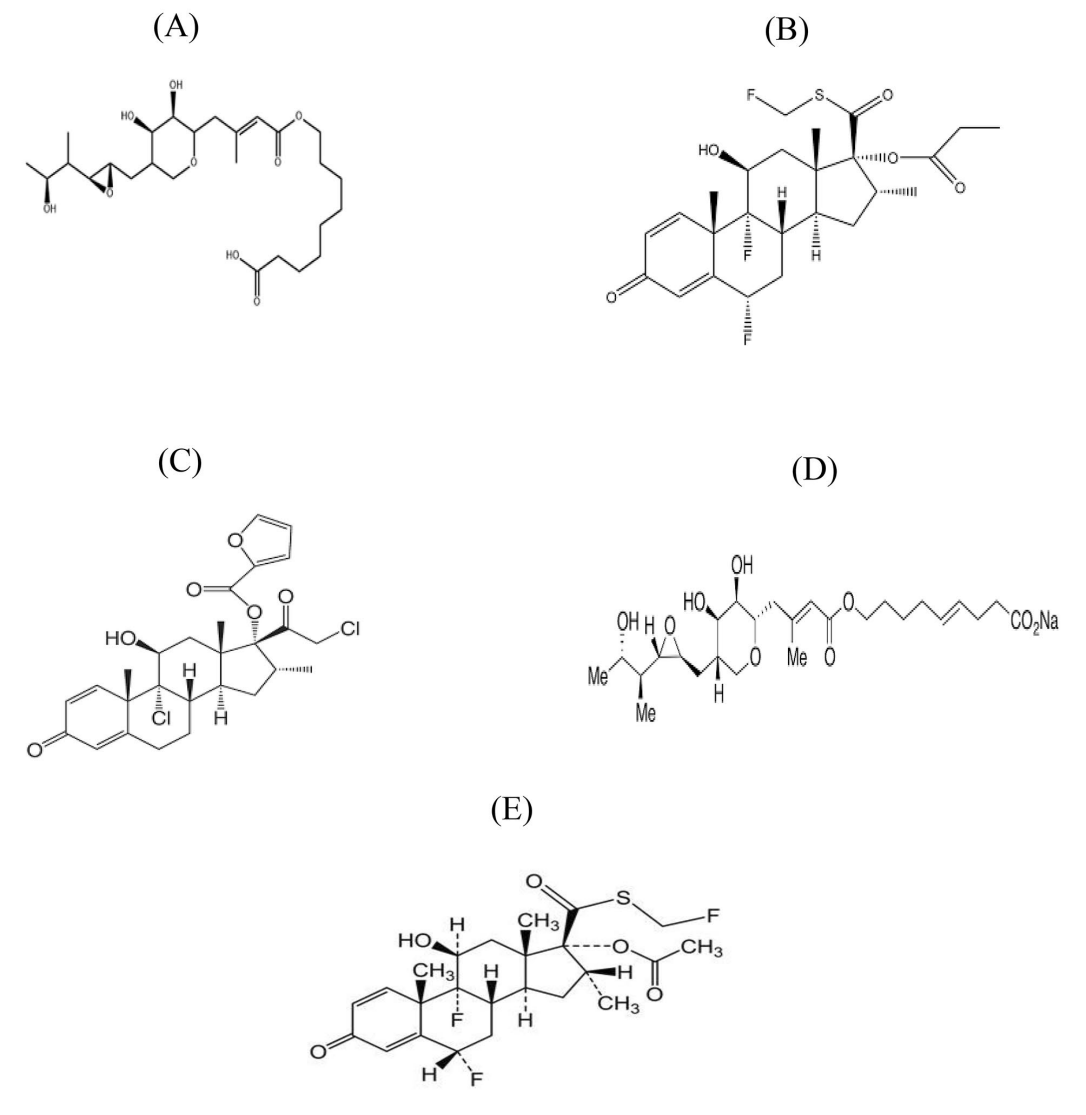
Chemical structures of (**A**) Mupirocin, (**B**) Fluticasone Propionate, (**C**) Mometasone furoate, (**D**) Pseudomonic acid-D and (**E**) Fluticasone impurity-C

Consequently, the aim of this study was to create two, simple and precise chromatographic methods, HPTLC-densitometry and RP-HPLC for the determination of MUP in its binary mixtures with FLU (mixture (1)) and MF (mixture (2)) at the same time with their impurities; Pseud-D and FIC (in mixture (1)) and Pseud-D (in mixture (2)) (Fig. 1). Both methods were validated in compliance with ICH recommendations and used to resolve the aforementioned components in laboratory-prepared mixtures and topical pharmaceutical formulations. The eco-friendliness of the two methods was investigated using the National Environmental Method Index, analytical Eco scale, Green Analytical Procedure Index and Analytical Greenness metric approaches.

## Experimental

### Instrumentation

#### HPTLC-densitometric method

Camag TLC scanner, model 3 S/N 1,302,319 (Camag, Switzerland) operated with win CATS® software, Linomat 5 auto sampler (Switzerland). Precoated HPTLC- plates (10 × 10 cm, 0.2 mm) silica gel 60 F_254_ (Merck, Germany).

#### HPLC method

Waters arc HPLC consisted of quaternary pump and PDA detector model 2998. The stationary phase was Agilent Eclipse XDB (250 mm×4.6 mm, 5 μm) C18 column (United States). pH-meter; Digital pH/MV/TEMP/ATC meter, Model- 5005, Jenco Instruments (California, USA).

### Materials and reagents

#### Chemicals and reagents

All chemicals utilized in this work were of analytical grade, and the solvents were of HPLC grade; toluene (Piochem, Egypt), chloroform (Fisher - Belgium), ethanol (Chem-Lab, Belgium), methanol (Chem-Lab, Belgium), glacial acetic acid (Piochem, Germany), Ortho-Phosphoric acid (Fischer Scientific, UK), distilled water (Milli-Q, USA), di-Sodium Hydrogen Phosphate (Emsure, Germany).

#### Authentic samples

MUP was purchased from Clearsynth Labs Limited, India, FLU was supplied by GlaxoSmithKline, England and MF was kindly supplied by Symbiotica group, Australia. Their purities were checked and found to be 99.7%, 99.2% and 98.8%; respectively according to the reported methods [12, 16].

Pseud-D and FIC were purchased from council of European Directorate for the Quality of Medicines and Health Care of the Council of Europe, India.

#### Pharmaceutical dosage form

Flutibact® skin Ointment (10gm), for atopic dermatitis, 2% w/w MUP / 0.005% w/w FLU, batch no.N1177, manufactured by Glaxo-SmithKline.

Metos-m® ointment (5gm), for atopic dermatitis, 2% w/w MUP / 0.1% w/w MF ointment, batch no. E1672 manufactured by West-Coast pharmaceutical works, ltd, India.

### Standard solutions

#### HPTLC-densitometry

Stock solution (8 mg. mL^− 1^) of MUP was prepared in methanol.

Stock solutions (1 mg. mL^− 1^) of FLU, MF, Pseud-D and FIC were prepared in methanol.

#### HPLC method

In methanol, stock solutions (1 mg. mL^-1^) of each of the aforementioned components were individually prepared.

### Chromatographic conditions

#### HPTLC-densitometry

The analysis was performed on HPTLC plates made of silica gel 60 F_254_ (10 × 10 cm, 0.2 mm) as the stationary phase. The mobile phase was toluene: chloroform: ethanol at ratio of (5:4:2, by volume), respectively. The plates were developed in a chromatographic tank at room temperature, ascendingly, after being saturated for 30 min. Using a Camag TLC scanner operating in absorbance mode, spots of the studied drugs were scanned at 220 nm for MUP detection and 254 nm for FLU, MF, Pseud-D and FIC detection.

#### HPLC method

HPLC analysis was performed using Agilent Eclipse XDB (250 mm×4.6 mm, 5 μm) C18 column as stationary phase. The mobile phase consisted of methanol: Sodium di-hydrogen phosphate (pH 3) applied in stepwise gradient elution with respective ratios of (50: 50 v/v, which turned to 80: 20 v/v) for mixture (1). Table 1 shows the stepwise gradient program that was used. For mixture (2), the same mobile phase was used in isocratic elution in the ratio (80: 20, v/v) respectively. The flow rate of the mobile phase was set to 1 mL/min. The pH of the mobile phases was adjusted to 3 using *ortho*-phosphoric acid. Analyses were carried out at ambient temperature and UV detection at 220 and 240 nm for mixture (1) and 220 and 248 nm for mixture (2). Each sample was analysed in three replicates with a 10 µL injection volume.

**Table 1:** Gradient programme of the HPLC method for determination of MUP, FLU, Pseud-D, and FIC

Time (min)	Buffer (%)	Methanol (%)	Flow rate(mL/min)
0–7 min	50%	50%	1mL/min
8–15 min	20%	80%	1mL/min
16–22 min	50%	50%	1mL/min

### Construction of calibration curves

#### HPTLC-densitometry method

Aliquots (0.25-5 mL) of MUP were accurately transferred from its stock standard solution (8 mg. mL^− 1^) into a set of 10-mL volumetric flasks and the volumes were completed to the mark with methanol to obtain a concentration range of (2–40 µg. band^− 1^). Aliquots (0.1-2 mL for FLU, 0.1-4 mL for MF, 0.2-4 mL for FIC and 0.1-2 mL for Pseud-D) were accurately transferred from their stock standard solutions (1 mg. mL^− 1^) into separate sets of 10-mL volumetric flasks and the volumes were completed to the mark with methanol to obtain concentration range of (0.1-2 µg. band^− 1^) for FLU, (0.1-4 µg. band^− 1^) for MF, (0.1-2 µg. band^− 1^) for Pseud-D and (0.2-4 µg. band^− 1^) for FIC. Ten µL were applied for each concentration on HPTLC plates using a Camag Linomat 5 automatic applicator. The chromatographic conditions described above were followed, and the plates were developed in a normal ascending manner. The regression parameters were calculated using calibration curves that show the relationship between the measured area under the peak and the equivalent drug concentration in micrograms per band.

#### HPLC method

Aliquots of MUP, FLU, MF, Pseud-D and FIC were transferred precisely from their standard solutions (1 mg. mL^− 1^) into a set of 10-mL volumetric flasks. The volumes were completed to the mark with methanol to obtain concentration range of (80–800 µg. mL^− 1^) of MUP, (0.5–50 µg. mL^− 1^) of FLU, (1–20 µg. mL^− 1^) of MF, (1–16 µg. mL^− 1^) of Pseud-D and (0.4–12 µg. mL^− 1^) of FIC. Triplicate injections were made for each concentration injected using an Agilent Eclipse XDB (250 mm×4.6 mm, 5 μm) C18 column at flow rate 1mL min^− 1^ and UV detection was done at 220 and 240 nm for mixture (1) and 220 and 248 nm for mixture (2). By plotting the peak area against the respective drug concentrations, calibration curves were created. The system suitability and validation parameters were calculated.

### Application to pharmaceutical preparations

Ten grams of Flutibact® ointment and five grams of Metos-M® ointment were weighed accurately and separately dissolved in volumetric flasks containing 25 mL methanol, heated in a water bath at ~ 50 º C till the ointment base melted, then it was sonicated for 10 min. The solution was then centrifuged for 15 min at 4000 rpm, the centrifugate was quantitatively transferred into 50- mL volumetric flasks and the volume was completed with methanol to reach a final concentration of (40 µg. band^− 1^ of MUP and 0.1 µg band^− 1^ of FLU in Flutibact® ointment and 20 µg. band^− 1^ MUP and 1. µg band^− 1^ MF in Metos-M® ointment). The solution was then analysed using HPTLC.

For HPLC, the same procedure was followed for the extraction of drugs from five grams of Flutibact® and Metos-M® ointments. The solution of Flutibact® ointment was then used for HPLC analysis of FLU at 5 µg. mL^− 1^. One mL of each previously prepared solution was transferred separately into 10-mL volumetric flasks, and the volume was completed to the mark with methanol to reach a final concentration of 200 µg. mL^− 1^ of MUP from each ointment solution and 10 µg. mL^− 1^ of MF. The samples were analysed using the optimized chromatographic conditions, and the drugs’ recoveries were calculated using regression equations.

## Results and discussion

The advancement of pharmaceuticals has resulted in a revolution in human health. These pharmaceuticals would only perform their function if they were free of impurities and administered in the proper amount. These pharmaceutical formulations may develop impurities at different stages of development, storage, and transportation, consequently, they are dangerous to administer. To ensure that drugs can be used safely, various chemical and instrumental methods are developed to ascertain the purity of both the API as well as the final dosage form [20].

HPLC and TLC combined with UV detection are well known to be the most commonly employed methods for organic complex mixture separation and determination [21]. Because of its well-established separation power and quantification sensitivity, HPLC has been approved by the pharmaceutical industry as a reliable technique in analytical chemistry. This fact enabled HPLC to gain acceptance in pharmacopoeias and propelled HPLC to the forefront of pharmaceutical analysis techniques. Selective detection and sensitive measurement of drugs in mixtures and complicated matrices were made possible by HPLC’s powerful separation and flexibility to be combined with different detectors [22].

Planar chromatographic techniques such as TLC, on the other hand, are regarded as viable alternatives in most pharmacopoeial monographs. High performance thin-layer chromatography (HPTLC) is a reliable, simple, fast and efficient quantitative analytical tool. HPTLC is a TLC-based analytical technique that has been improved to enhance the resolution of the compounds to be separated as well as to allow their quantitative analysis. Some of the improvements, such as using higher-quality TLC plates with finer particle sizes in the stationary phase, enable better resolution. HPTLC is one of the chromatographic techniques available for constituent identification, impurity identification and determination, and active substance quantification. As a result, when compared to standard TLC, HPTLC is one of the best TLC techniques for analytical purposes due to its increased accuracy, reproducibility, and ability to document the results [23].

In this work, HPTLC- densitometry and HPLC methods are described for the simultaneous determination of MUP, FLU together with two of their impurities; Pseud-D and FIC in mixture (1) and MUP, MF and Pseud-D in mixture (2).

### Methods development

#### HPTLC-densitometry

**Fig. 2:**
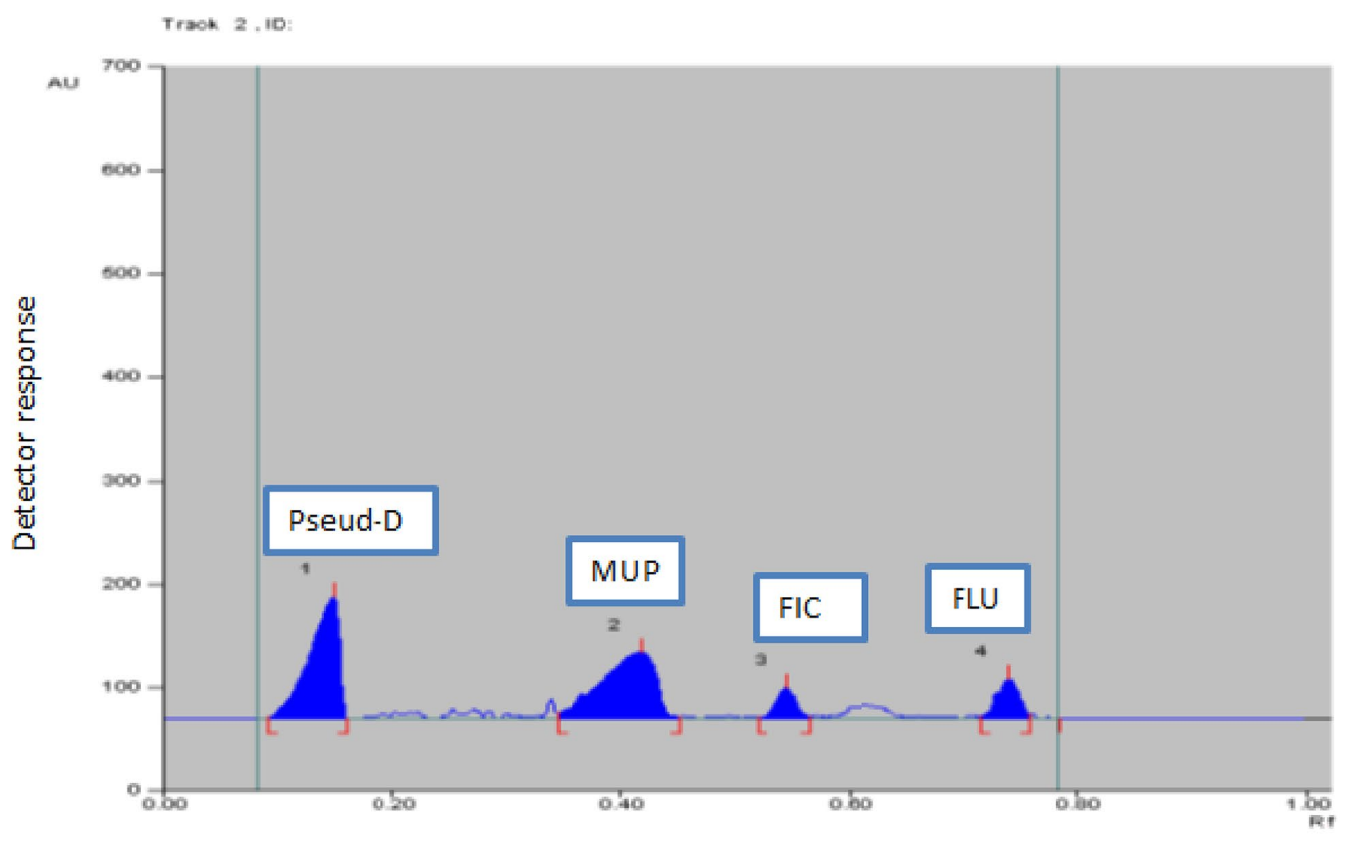
2D-TLC chromatogram of Pseud-D (1.6 µg/ band) (R_f_ = 0.15), MUP (40 µg/ band) (R_f_ = 0.42), FIC (0.1 µg/ band) (R_f_ = 0.55) and FLU (0.1 µg/ band) (R_f_ = 0.74), using a mobile phase of toluene: chloroform: ethanol (5:4:2, by volume) and detection at 254 nm

**Fig. 3:**
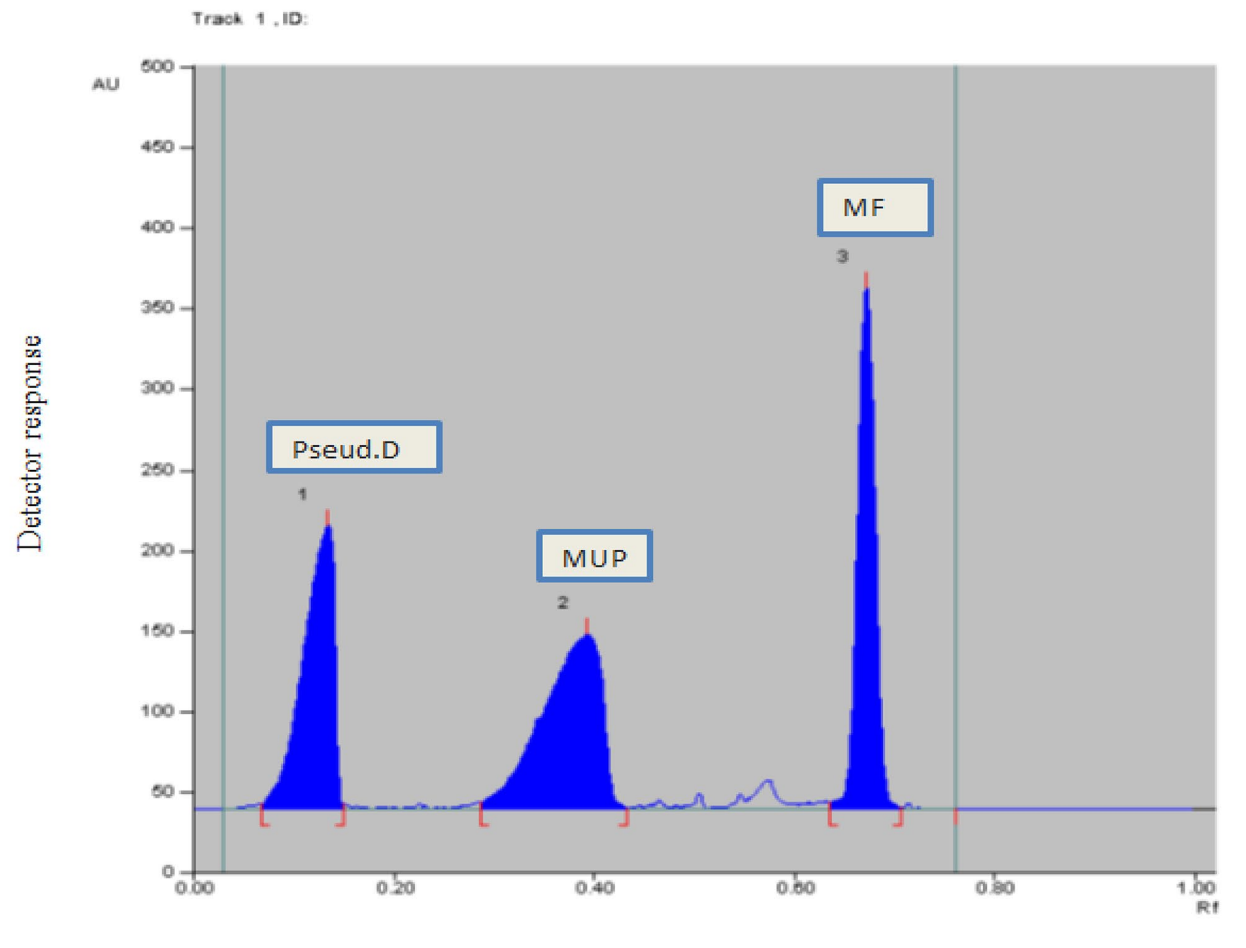
2-DTLC chromatogram of Pseud-D (0.8 µg/ band) (R_f_ = 0.14), MUP (20 µg/ band) (R_f_ = 0.39) and MF (1 µg/ band) (R_f_ = 0.68) using a mobile phase of toluene: chloroform: ethanol (5:4:2, by volume) and detection at 254 nm

Several trials were carried out in order to determine the optimal HPTLC-densitometric conditions. As a developing system, various ratios of n-heptane, methanol, ethyl acetate, and glacial acetic acid were first tried. However, n-heptane resulted in insufficient separation of MUP and FIC; additionally, by using methanol, Pseud-D separation was poor as it used to appear at the solvent front. By replacing n-heptane with toluene, good separation of MUP and FIC was obtained. Despite the toxicity and health hazards of chloroform, it gave better resolution of the cited components compared to methanol and ethyl acetate. The best separation of the cited components was achieved by toluene: chloroform: ethanol at a ratio of (5:4:2, by volume). The chromatograms were scanned at different wavelengths to select the best wavelength for quantitation. Scanning was carried out at λ_max_ of each compound as well as at 254 nm (universal wavelength). For MUP quantitation, it was found that working at its λ_max_ 220 nm gave better sensitivity than 254 nm. As for the remaining components, comparable results were obtained upon using either λ_max_ or the universal wavelength 254 nm. Therefore, for ease of application, the remaining components were scanned at 254 nm. The R_f_ values were 0.15 ± 0.02, 0.42 ± 0.02, 0.55 ± 0.02 and 0.74 ± 0.02 for Pseud-D, MUP, FIC and FLU, respectively (Fig. 2). For mixture (2), the R_f_ values were 0.14 ± 0.02, 0.39 ± 0.02 and 0.68 ± 0.02 for Pseud-D, MUP, MF respectively (Fig. 3).

The performance of the current chromatographic conditions was assessed using system suitability parameters. Good results regarding selectivity, resolution and tailing factors were attained (Table 2) as compared to the reference values [24].

**Table 2:** Parameters required for system suitability tests of the proposed HPTLC method

Parameters	Mixture (1)				Mixture (2)			Reference values [24]
	Pseud-D	MUP	FIC	FLU	Pseud-D	MUP	MF	
Retardation factor (R_f_ )	0.15	0.42	0.55	0.74	0.14	0.39	0.68	
Resolution (Rs)	2.18	1.52	3.8		1.8	2.32		Rs > 1.5
Tailing factor (T)	0.83	0.95	0.83	0.84	0.8	1	0.83	
Retention factor (k’) ^a^	5.66	1.38	0.818	0.35	6.14	1.56	0.47	1–5
Selectivity factor (α)	4.10	1.68	2.33		3.93	3.32		α > 1
Column efficiency (N) ^b^	1024	711	4096	3505	1600	256	4096	
Height equivalent to theoretical plate (HETP) (mm)	0.0007	0.001	0.0001	0.0002	0.0005	0.0031	0.0002	The smaller the value, the higher the column efficiency

^a^*α* = *k*2/*k*1, where *k* is the capacity factor: *k* = (1 − *R*_f_)/*R*_f_.^b^*R*s = [2 (*R*f2 − *R*f1)]/ (*W*1 + *W*2), where *R*f is retardation factor and *W* is peak width at 5% from the baseline of the peak height

^C^*N* = 16 (*z*/*w*) ^2^, where *z* is the migration length of the spot and *w* is the spot width

#### High-performance liquid chromatography

**Fig. 4:**
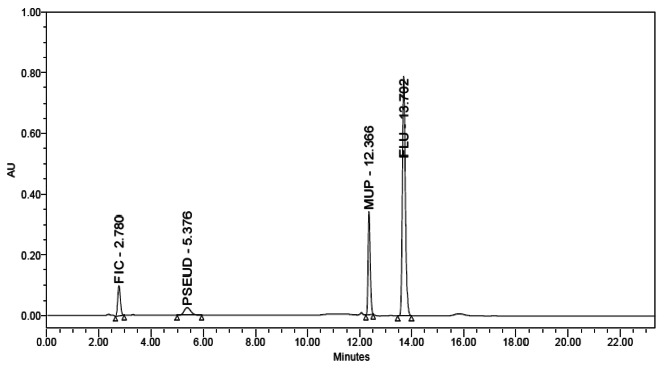
HPLC chromatogram of FIC (1 µg. mL^− 1^) (t_R_=2.78), Pseud–D (8 µg. mL^− 1^) (t_R_=5.37), MUP (200 µg. mL^− 1^) (t_R_ =12.36) and FLU (5 µg. mL^− 1^) (t_R_ = 13.70) using Agilent Eclipse XDB C18 column (250 mm×4.6 mm, 5 μm) with a mobile phase of linear gradient elution

**Fig. 5:**
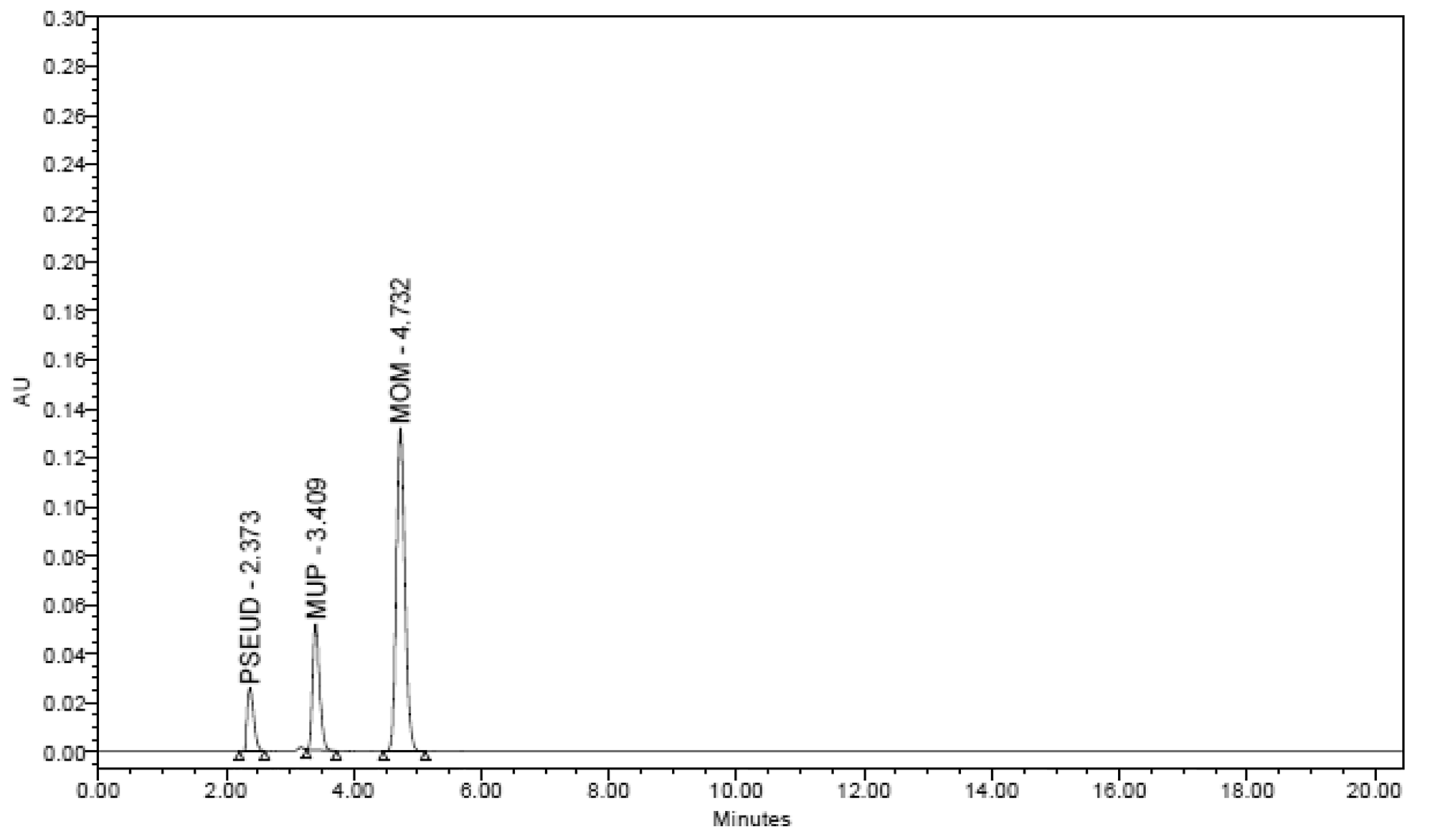
HPLC chromatogram of Pseud–D (8 µg. mL^− 1^) (tR = 2.37), MUP (200 µg. mL^− 1^) (tR = 3.40) and MF (10 µg. mL^− 1^) (tR = 4.73) using Agilent Eclipse XDB C18 column (250 mm×4.6 mm, 5 μm) with a mobile phase of linear isocratic elution

For optimization and development, an Agilent Eclipse XDB (250mm4.6 mm, 5 m) C18 column was used. Numerous mobile phases of phosphate buffer, methanol and acetonitrile were examined at various ratios, pH levels and flow rates. Good chromatographic separation was achieved by using a mobile phase consisting of methanol: sodium di-hydrogen phosphate (pH 3) applied in stepwise gradient mode starting with respective ratios of 50:50 then switching to 80:20 for mixture (1) and isocratic elution with ratio of (80: 20, v/v) for mixture (2). The mobile phase was pumped at a rate of 1 mL/min. Total runtime was 15 min. UV detection was carried out at λ_max_ for each component as it gave better sensitivity (220 and 240 nm for mixture (1) and 220 and 248 nm for mixture (2)). The obtained t_R_ values were 2.78, 5.37, 12.36 and 13.70 min for FIC, Pseud-D, MUP and FLU, respectively in mixture (1) (Fig. 4) and 2.37, 3.40 and 4.73 min for Pseud-D, MUP, MF, respectively in mixture (2) (Fig. 5).

System suitability testing was used to evaluate the operating HPLC conditions, and the outcomes were satisfactory (Table 3) as compared to the reference values [25].

**Table 3:** Parameters required for system suitability tests of the proposed HPLC method

Parameters	Mixture (1)				Mixture (2)			Reference value [25]
	FIC	Pseud-D	MUP	FLU	Pseud-D	MUP	MF	
Retention time (t_R_)	2.78	5.37	12.36	13.70	2.37	3.40	4.73	
Resolution (Rs)	7.77	22.1	7.16		5.34	5.81		≥ 1.5
Tailing factor (T)	1.11	1.18	1.23	1.20	1.18	1.17	1.18	T = 1 for typical symmetric peak
Retention factor (k)	1.05	2.98	8.15	9.14	2.85	1.52	2.5	1–5
Selectivity factor (α)	2.81	2.73	1.12		2.01	1.64		> 1
Column efficiency (N)	3270	2110	101,000	75,100	2604	4779	67,600	Increases with efficiency of the separation
Height equivalent to theoretical plates (HETP)	0.0076	0.0118	0.00024	0.00033	0.0096	0.0052	0.0003	The smaller the value, the higher the column efficiency

### Methods validation

According to ICH criteria, the two proposed methods’ linearity, accuracy, precision, specificity and robustness were all validated [6].

#### Linearity and range

High degree linearity for HPTLC method was achieved in concentration ranges of (2–40 µg.band^− 1^) for MUP, (0.1-2 µg.band^− 1^) for FLU, (0.1-4 µg.band^− 1^) for MF, (0.1-2 µg.band^− 1^) for Pseud-D and (0.2-4 µg.band^− 1^) for FIC. The concentration ranges of (80–800 µg. mL^− 1^) for MUP, (0.5–50 µg. mL^− 1^) for FLU, (1–20 µg. mL^− 1^) for MF, (1–16 µg. mL^− 1^) for Pseud-D, and (0.4–12 µg. mL^− 1^) for FIC were utilized to form the linearity for HPLC method (Tables 4 and 5).

**Table 4:** Parameters and validation sheet for determination of the cited compounds by the proposed HPTLC method

Parameter	HPTLC method				
	MUP	FLU	MF	Pseud-D	FIC
Linearity range	2–40 µg/band	0.1-2 µg/band	0.1-4 µg/band	0.1-2 µg/ band	0.2-4 µg/ band
Accuracy Mean ± RSD	101.29± 0.677	99.59 ± 1.146	98.76 ± 1.670	100.61 ± 0.519	99.80 ± 1.447
Correlation coefficient (r)	0.9992	0.9997	0.9999	0.9999	0.9998
Slope	1399.3	3449.4	2997.8	6338.4	872.69
Standard error [SE] of slope	114.31	148.45	211.89	25.36	6.57
Intercept	4403.9	765.75	1961.5	777.19	26
Standard error [SE] of intercept	2504.61	179.99	384.08	23.63	12.53
Precision Repeatability ^a^ (%RSD)	1.788	0.718	1.475	1.018	0.516
Intermediate precision ^b^ (%RSD)	1.616	1.00	0.968	1.119	1.159
Robustness* (RSD %)	0.183	1.23	0.633	0.383	1.508
LOD	0.358 µg/band	0.006 µg/ band	0.012 µg/ band	0.030 µg/ band	0.029 µg/ band
LOQ	1.086 µg/ band	0.020 µg/ band	0.037 µg/ band	0.091 µg/ band	0.089 µg/ band

^a^ average of three different concentrations repeated three times within the day

^b^ average of three different concentrations repeated three times in three successive days

*Average of three determinations

**Table 5:** Parameters and validation sheet for determination of the cited compounds by the proposed HPLC method

Parameter	Mixture (1)				Mixture (2)		
	MUP	FLU	Pseud-D	FIC	MUP	MF	Pseud-D
Linearity range	80–800 µg/mL	0.5–50 µg/mL	1–16 µg/mL	0.4–12 µg/mL	80–800 µg/mL	1–20 µg/mL	1–16 µg/mL
Accuracy Mean ± RSD	98.72± 1.772	100.11 ± 0.688	98.93 ± 1.335	99.72 ± 0.634	99.08± 1.75	100.78 ± 0.708	99.60 ± 0.769
Correlation coefficient (r)	0.9995	0.9999	0.9991	0.9997	0.9997	0.9997	0.9999
Slope	13,525	25,749	60,486	28,757	13,530	25,388	60,509
Standard error [SE] of slope	137.27	110.29	890.66	265.98	110.13	209.06	351.25
Intercept	279,718	5790	13,149	408.31	237,931	471.67	21,505
Standard error [SE] of intercept	58961.25	2648.21	8267.73	1881.27	47305.33	2350.78	3260.52
Precision Repeatability ^a^ (%RSD)	1.38	1.00	2.00	1.22	1.816	1.411	1.167
Intermediate precision ^b^ (%RSD)	0.679	0.947	1.51	1.28	1.90	1.382	1.212
LOD	7.208 µg/mL	0.087 µg/mL	0.322 µg/ mL	0.014 µg/ mL	3.449 µg/mL	0.016 µg/mL	0.068 µg/ mL
LOQ	21.842 µg/mL	0.265 µg/ mL	0.976 µg/ mL	0.044 µg/ mL	10.452 µg/mL	0.05 µg/ mL	0.210 µg/ mL

^a^ average of three different concentrations repeated three times within the day

^b^ average of three different concentrations repeated three times in three successive days

#### Precision

The precision was assessed by analyzing three distinct concentrations in triplicates on the same day (for repeatability) and on three subsequent days (for intermediate precision). For HPTLC, the analyzed concentrations were (2, 8, 20 µg. band^− 1^) for MUP, (0.2, 0.6, 1.6 µg. band^− 1^) for FLU, (0.4, 0.8, 2 µg. band^− 1^) for MF and (0.4, 0.8, 2 µg. band^− 1^) for Pseud-D and (0.8, 1, 2 µg. band^− 1^) for FIC. For HPLC, the analyzed concentrations were (80, 200, 400 µg. mL^− 1^) for MUP, (1, 10, 20 µg. mL^− 1^) for FLU, (4, 8, 20 µg. mL^− 1^) for MF, (4, 8, 12 µg. mL^− 1^) for Pseud-D and (4, 6, 10 µg. mL^− 1^) for FIC. Relative standard deviation % were then estimated and were found to be less than 2% as shown in Tables 4 and 5.

#### Accuracy

Different concentrations of the analyzed drugs and their impurities were determined to assure the accuracy of the developed methods. The related regression equations were used to determine the concentrations, and mean recoveries were computed. Obtained mean percentage recoveries were satisfactory as shown in Tables 4 and 5.

#### Robustness

Robustness of HPTLC method was assessed by applying deliberate variations in the ratio of the developing system with a value of ± 0.1 (Table 4). The robustness of HPLC method was assessed by altering flow rate (± 0.1) and the mobile phase composition. There were no significant changes in the system suitability parameters, and obtained RSD% values were satisfactory (Supplementary Tables S1 and S2).

#### Specificity

Good separation achieved between the studied compounds assured the methods’ specificity as shown in Figs. 2, 3, 4 and 5.

#### LOD and LOQ

Values of LOD and LOQ for the cited compounds were calculated as per ICH recommendations. LOD was calculated as (3.3 × SD of the response/slope) while LOQ= (10 × SD of the response/slope) (Tables 4 and 5).

### Application to commercial ointments

The suggested methods were applied to determine MUP and FLU in Flutibact® ointment and MUP and MF in Metos-M® ointment. The results demonstrated good recovery in accordance with the labeled amounts (Supplementary Table S3). Various amounts of MUP, FLU, and MF pure standards were added to aliquots of topical formulations and analyzed in triplicates using the standard addition technique. Satisfactory recoveries were obtained as demonstrated in (Supplementary Table S3).

### Statistical analysis

Statistical investigation was dedicated to contrast the results achieved by applying the suggested methods for analyzing the pure forms of the cited drugs to the previously reported methods for determining MUP and FLU [12] and MUP and MF [16]. The values for the mean and variance were compared using the calculated t-test and F-test, (Supplementary Tables S4 and S5). Values obtained were lower than their corresponding theoretical ones revealing insignificant differences between the proposed and reported methods. Furthermore, none of the reported methods addressed the determination of any of the cited drugs’ impurities. As a result, our proposed methods outperform others in determining MUP, FLU, and two of their impurities: Pseud-D and FIC in mixture (1) and MUP, MF, and Pseud-D in mixture (2).

## Green Profile Assessment Metrics

To assess the greenness profile of the proposed methods (compared to the reported methods); four monitoring well-organized green metrics were chosen; a qualitative tool, the “National Environmental Method Index” (NEMI) and a semi-quantitative tool, the “Analytical Eco-Scale” method, the “Green Analytical Procedure Index” (GAPI) and Analytical Greenness metric (AGREE).

NEMI labeling is one of the oldest tools used to assess the greenness of analytical procedures. This method addresses four requirements: (1) none of the chemicals should be present in the persistent, bio accumulative and toxic chemicals list, (2) none of the chemicals should be listed in D, F, P or U hazardous wastes lists, (3) the pH of the sample should be within 2–12 range and (4) less than 50 g of waste per sample should be produced [26]. The proposed RP-HPLC method, which uses methanol and sodium di-hydrogen phosphate buffer (pH 3) with waste less than 50 g per sample; met all the four requirements, so all the four quadrants of NEMI pictogram were shaded green (Supplementary Table S6), whereas the proposed HPTLC method failed to meet the hazard quadrant due to the presence of chloroform in the U and F lists but met the remaining requirements (Supplementary Table S7).The reported HPLC methods for determination of MUP / FLU and MUP / MF showed only three green quadrants. Therefore, the two proposed methods are considered green with respect to NEMI. (Supplementary Tables S8 and S9)

The Eco-Scale methodology relies on subtracting penalty points from a base of 100 for used solvents and method parameters. A score of more than 75 on the analytical eco-scale is considered an excellent green method [27]. The analytical Eco scale scores of the proposed HPLC and HPTLC-densitometric methods were found to be 87 and 84, respectively (Supplementary Tables S6 and S7), the reported method for the determination of MUP/ FLU and MUP/ MF scored 89 (Supplementary Tables S8 and S9). Our proposed methods are considered excellent green and displayed superior green profiles. Furthermore, they were able to quantify the APIs (MUP, MF, and FLU) concurrently with their impurities, making them superior to the published methods.

The GAPI green assessment evaluates the green profile of the full analytical procedure from sample preparation to final determination steps [28]. The GAPI employs five pentagrams to evaluate and quantify each step of the analysis process using a colour code; green, yellow, and red which represent the method’s environmental impact as low, medium, and high, respectively. The GAPI pentagram of the proposed RP-HPLC and HPTLC- densitometric methods revealed that each has minimal environmental impact compared to the reported methods. RP-HPLC method achieved larger numbers of green fields (12), (4) yellow fields and no red fields. The proposed HPTLC- densitometric method achieved (13) green fields (3) yellow fields with no red field while the reported methods achieved (11) green fields, (5) yellow fields and no red fields (Supplementary Tables S6, S7, S8 and S9).

The Analytical Greenness metric assessment criteria are taken from the 12 principles of green analytical chemistry (SIGNIFICANCE) and are transformed into a unified 0–1 scale. The result is a pictogram indicating the final score, performance of the analytical procedure in each criteria, and weights assigned by the user [29]. According to Analytical Greenness metric, the greenness comparison indicated the superiority of both of our proposed methods over the reported methods as the score of the proposed RP-HPLC method was 0.81 (Supplementary Table S6), and that of the proposed HPTLC- densitometric method was 0.79 (Supplementary Table S7), while the reported method for the determination of MUP/ FLU and that of the determination of MUP/ MF scored 0.67 and 0.66, respectively (Supplementary Tables S8 and S9).

## Conclusion

Two chromatographic techniques, HPLC and HPTLC, were developed for determining some topically administered drugs namely; MUP, FLU and MF together with their impurities Pseud-D and FIC. The methods have been validated for accuracy, precision, specificity, and robustness in accordance with ICH guidelines. The proposed HPTLC-densitometric method is straightforward, with advantages such as short analysis time, lower cost per analysis, and good component resolution. The HPLC technique has the advantage of separating component in a relatively short time with accepted resolution. Greenness assessment was carried out using NEMI, Analytical Eco-scale, GAPI and AGREE approaches and accepted results were obtained. All of the proposed methods can be used in routine analysis and quality control laboratories to determine intentional drugs in pure form and in pharmaceutical formulations.

### Electronic supplementary material

Below is the link to the electronic supplementary material.

Supplementary Material 1

## Data Availability

Datasets generated and/or analyzed during the current study are available from the corresponding author on reasonable request.
