# Supplementary material for: Greenness assessment of two chromatographic methods developed for the determination of Mupirocin in two binary mixtures along with its impurity
Source: BMC Chem. 2023 Oct 27;17(1):145. doi: 10.1186/s13065-023-01055-5 (PMC10612294; doi:10.1186/s13065-023-01055-5)
Supplement: Supplementary file 1 — Supplementary Material 1 [file 13065_2023_1055_MOESM1_ESM.docx]

**Supplementary Material**

**For**

**Greenness assessment of two chromatographic methods developed for the determination of mupirocin in two binary mixtures along with its impurity**

**Supplementary Table (S1)**: Robustness of the proposed HPLC method for the determination of MUP, FLU, Pseud-D and FIC

| Parameter | | Resolution | | | | | | Retention factor k | | | | Selectivity factor α | | | | | |
| --- | --- | --- | --- | --- | --- | --- | --- | --- | --- | --- | --- | --- | --- | --- | --- | --- | --- |
| Flow rate |  | FIC | Pseud-D | | MUP | | FLU | FIC | Pseud-D | MUP | FLU | FIC | Pseud-D | | MUP | | FLU |
|  | 1.1 | 7.62 | | 23 | | 7.023 | | 1.07 | 3 | 8.76 | 9.75 | 2.8 | | 2.91 | | 1.11 | |
|  | 0.9 | 7.97 | | 19.8 | | 7.4 | | 1.059 | 3.01 | 7.6 | 8.59 | 2.84 | | 2.52 | | 1.13 | |
|  | 1 | 7.9 | | 21.9 | | 7.18 | | 1.05 | 3.01 | 8.16 | 9.15 | 2.84 | | 2.7 | | 1.12 | |
|  | SD | 0.185 | | 1.62 | | 0.189 | | 0.01 | 0.005 | 0.58 | 0.58 | 0.023 | | 0.195 | | 0.01 | |
| Mobile phase ratio | Gradient 1 | 7.9 | | 21.9 | | 7.18 | | 1.05 | 3.01 | 8.16 | 9.15 | 2.84 | | 2.7 | | 1.12 | |
|  | Gradient 2 | 5.9 | | 23.5 | | 9.6 | | 0.966 | 1.69 | 10.4 | 10.1 | 1.75 | | 3.65 | | 1.86 | |
|  | SD | 1.414 | | 1.13 | | 1.71 | | 0.059 | 0.933 | 1.58 | 0.67 | 0.77 | | 0.671 | | 0.52 | |

**Supplementary Table (S2)**: Robustness of the proposed HPLC method for the determination of MUP, MF and Pseud-D

| Parameter | | Resolution | | | | Retention factor k | | | Selectivity factor α | | | |
| --- | --- | --- | --- | --- | --- | --- | --- | --- | --- | --- | --- | --- |
| Flow rate |  | Pseud-D | MUP | | MF | Pseud-D | MUP | MF | Pseud-D | MUP | | MF |
|  | 1.1 | 4.46 | | 5.51 | | 7.54 | 1.52 | 2.51 | 2.025 | | 1.643 | |
|  | 0.9 | 4.35 | | 5.66 | | 7.43 | 1.5 | 2.47 | 2.027 | | 1.644 | |
|  | 1 | 5.34 | | 5.81 | | 7.57 | 1.52 | 2.5 | 2.013 | | 1.643 | |
|  | SD | 0.54 | | 0.15 | | 0.07 | 0.011 | 0.02 | 0.007 | | 0.0005 | |
| Mobile phase ratio | Linear  20-80 | 5.34 | | 5.81 | | 7.57 | 1.52 | 2.5 | 2.013 | | 1.643 | |
|  | Gradient  (0-7)  50-50  (7.5-15) 20-80 | 7.9 | | 7.43 | | 5.85 | 4.12 | 5.14 | 2.85 | | 1.124 | |
|  | SD | 1.81 | | 1.14 | | 1.126 | 1.83 | 1.86 | 0.591 | | 0.366 | |

**Supplementary Table (S3)**: Determination of MUP, FLU and MF in their dosage forms and application of standard addition technique using the proposed methods

| Pharmaceutical  formulation | Drug | Method | %Found ^*^  ± RSD | Claimed taken  ( HPTLC μg.band^-1^), (HPLC μg.mL^-1^) | Standard added  ( HPTLC μg.band^-1^), (HPLC μg.mL^-1^) | Mean^*^ ± RSD% |
| --- | --- | --- | --- | --- | --- | --- |
| Flutibact^®^  B.N. N1177 | MUP | HPTLC | 97.41± 0.930 | 2 | 1 | 100.23±0.741 |
|  |  |  |  |  | 2 |  |
|  |  |  |  |  | 4 |  |
|  | FLU |  | 98.73± 1.610 | 0.1 | 0.5 | 99.17±0.898 |
|  |  |  |  |  | 0.7 |  |
|  |  |  |  |  | 0.9 |  |
|  | MUP | HPLC | 96.88±1.510 | 200 | 100 | 97.87±1.993 |
|  |  |  |  |  | 200 |  |
|  |  |  |  |  | 300 |  |
|  | FLU |  | 96.47±1.041 | 5 | 5 | 100.21±0.670 |
|  |  |  |  |  | 10 |  |
|  |  |  |  |  | 15 |  |
| Metos-M^®^  B.N. E1672 | MUP | HPTLC | 100.80±1.926 | 2 | 1 | 99.81±1.396 |
|  |  |  |  |  | 2 |  |
|  |  |  |  |  | 4 |  |
|  | MF |  | 98.80± 1.974 | 0.1 | 0.1 | 99.07±1.170 |
|  |  |  |  |  | 0.2 |  |
|  |  |  |  |  | 0.4 |  |
|  | MUP | HPLC | 98.21± 0.437 | 200 | 100 | 99.10±1.990 |
|  |  |  |  |  | 200 |  |
|  |  |  |  |  | 300 |  |
|  | MF |  | 99.16± 1.360 | 10 | 2 | 99.89±0.569 |
|  |  |  |  |  | 4 |  |
|  |  |  |  |  | 6 |  |

*Average of three determinations

**Supplementary Table (S4):** Statistical comparison for the results obtained by the proposed methods and the reported method for the determination of MUP and FLU in their pure forms

| parameter | MUP | | FLU | | MUP | | FLU | |
| --- | --- | --- | --- | --- | --- | --- | --- | --- |
|  | HPTLC | Reported method ^**[12]^ | HPTLC | Reported method ^**[12]^ | HPLC | Reported method^**^ ^[12]^ | HPLC | Reported method^**^ ^[12]^ |
| Mean of recoveries | 101.25 | **99.51** | 99.59 | **99.70** | 98.72 | **99.51** | 100.10 | **99.70** |
| SD | 0.712 | **1.58** | 1.14 | **0.893** | 1.749 | **1.58** | 0.689 | **0.893** |
| Variance | 0.507 | **2.49** | 1.30 | **0.798** | 3.06 | **2.49** | 0.474 | **0.798** |
| n | 9 | 9 | 9 | 9 | 9 | 9 | 9 | 9 |
| F-test | 1.97 (3.43)* | ------ | 1.63 (3.43)* | ------ | 1.214 (3.43)* | ------ | 1.68 (3.43)* | ------ |
| Student’s t-test | 1.72 (1.78)* | ------ | 0.183 (1.77)* | ------ | 1.003 (2.11)* | ------ | 1.06 (2.13)* | ------ |

***** Theoretical value for t-test and F-ratio for P = 0.05.

^[12]**^ HPLC method, C18 column syncronis (250 × 4.6 mm, 5 μm). Using a mobile phase consisting of 0.01% OPA: Acetonitrile (30:70v/v) (pH: 5) pumped at a rate of 1.0 mL/min at 232 nm.

**Supplementary Table (S5):** Statistical comparison for the results obtained by the proposed methods and the reported method for the determination of MUP and MF in their pure forms

| parameter | MUP | | MF | | MUP | | MF | |
| --- | --- | --- | --- | --- | --- | --- | --- | --- |
|  | HPTLC | Reported method^**^ ^[16]^ | HPTLC | Reported method^** [16]^ | HPLC | Reported method^** [16]^ | HPLC | Reported method^**^ ^[16]^ |
| Mean of recoveries | 101.25 | **100.31** | 98.91 | **100.05** | 99.07 | **100.31** | 100.78 | **100.05** |
| SD | 0.71 | **1.002** | 1.47 | **1.15** | 1.85 | **1.002** | 0.778 | **1.15** |
| Variance | 0.504 | **1.004** | 2.16 | **1.335** | 3.03 | **1.004** | 0.511 | **1.335** |
| n | 9 | 9 | 9 | 9 | 9 | 9 | 9 | 9 |
| F-test | 1.98 (3.43)* | ------ | 1.62 (3.43)* | ------ | 2.36 (3.43)* | ------ | 2.61 (3.43)* | ------ |
| Student’s t-test | 1.72 (2.17)* | ------ | 1.24 (2.16)* | ------ | 0.851 (2.14)* | ------ | 1.60 (2.16)* | ------ |

**^*^** Theoretical value for t-test and F-value for p = 0.05.

^[16]**^ HPLC method, reversed-phase Phenomenax-luna C18 column using a mobile phase consisting of acetonitrile: Sodium di-hydrogen phosphate buffer (pH 6.8) (70:30 v/v) at a flow rate of 1 mL/min and UV detection at 240 nm.

**Supplementary Table (S6):** Greenness profiles of the proposed RP-HPLC method using different assessment tools

| **Method item Penalty points** | | **Greenness tools** |
| --- | --- | --- |
| Reagents  Methanol  sodium di-hydrogen phosphate  Ultrapure Water | 6  1  0  ∑7 | **NEMI**:  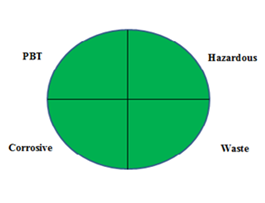 |
| Instrument  Energy ≤0.1 kWh per sample)  Occupational hazard  Waste (>10 mL) | 1  0  5  ∑5 | **GAPI:**  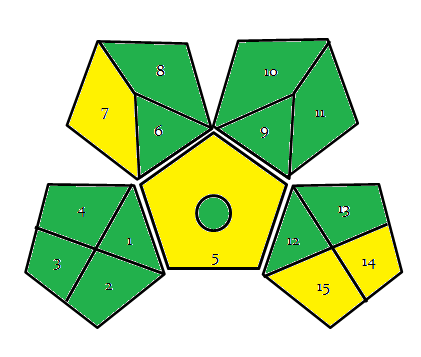 |
| Total penalty points  Analytical Eco-scale score  Analytical eco-scale score ≥75Excellent green method ≥50Acceptable green method  <50 Inadequate green method | 13  **87** | **AGREE:**  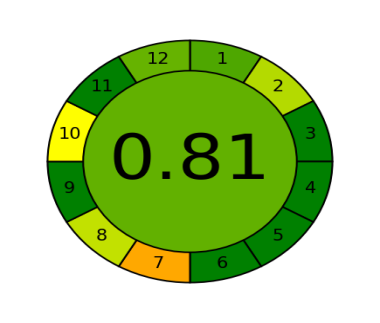 |

**Supplementary Table (S7):** Greenness profiles of the proposed HPTLC-densitometric method using different assessment tools

| **Method item Penalty points** | | **Greenness tools** |
| --- | --- | --- |
| Reagents  Toluene  Chloroform  Ethanol | 6  2  4  ∑12 | **NEMI**:  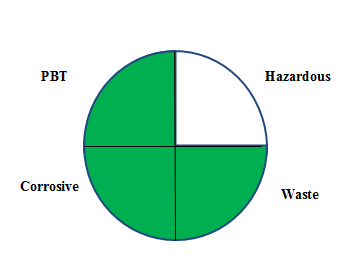 |
| Instrument  Energy (<0.1 kWh per sample) Occupational hazard  Waste (reused) | 1  0  3  ∑4 | **GAPI:**  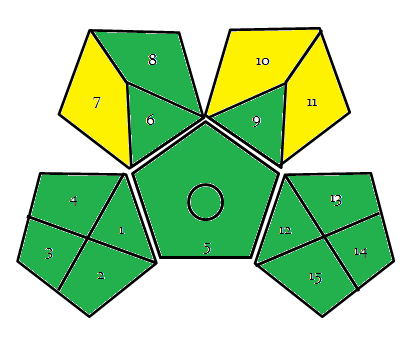 |
| Total penalty points  Analytical Eco-scale score  Analytical eco-scale score ≥75Excellent green method ≥50Acceptable green method  <50 Inadequate green method | 16  **84** | **AGREE:**  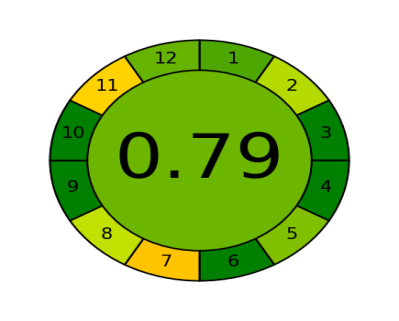 |

**Supplementary Table (S8):** Greenness profiles of the reported HPLC method for the determination of MUP and FLU^[12]^ using different assessment tools

| **Method item Penalty points** | | **Greenness tools** |
| --- | --- | --- |
| Reagents  Acetonitrile  sodium di-hydrogen phosphate  Ultrapure Water | 6  1  0  ∑7 | **NEMI**:  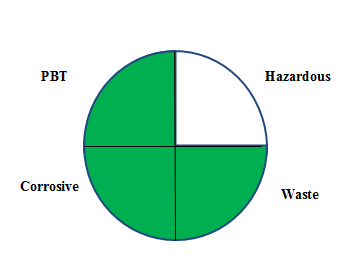 |
| Instrument  Energy (< 0.1kWh per sample)  Occupational hazard  Waste (1–10 ml) | 1  0  3  ∑4 | **GAPI:**  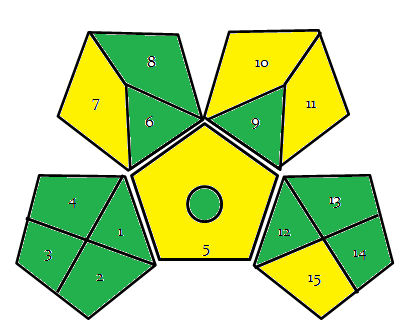 |
| Total penalty points  Analytical Eco-scale score  Analytical eco-scale score ≥75Excellent green method ≥50Acceptable green method <50 Inadequate green method | 11  **89** | **AGREE:**  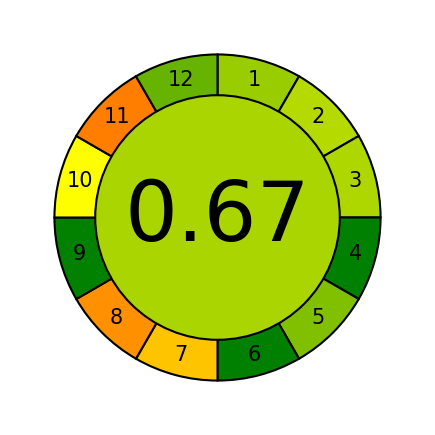 |

**Supplementary Table (S9):** Greenness profiles of the reported HPLC method for the determination of MUP and MF^[16]^ using different assessment tools

| **Method item Penalty points** | | **Greenness tools** |
| --- | --- | --- |
| Reagents  Acetonitrile  sodium di-hydrogen phosphate  Ultrapure Water | 6  1  0  ∑7 | **NEMI**:  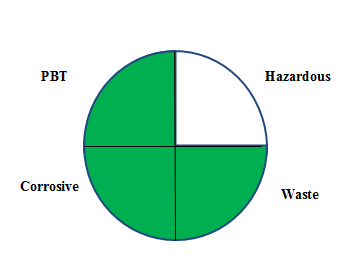 |
| Instrument  Energy (< 0.1kWh per sample)  Occupational hazard  Waste (1–10 ml) | 1  0  3  ∑4 | **GAPI:**  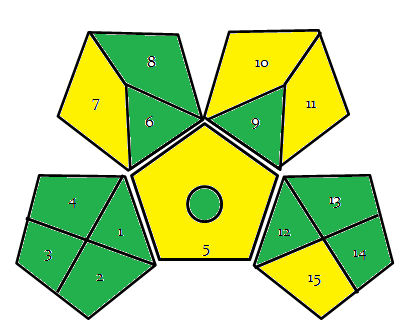 |
| Total penalty points  Analytical Eco-scale score  Analytical eco-scale score ≥75Excellent green method ≥50Acceptable green method  <50 Inadequate green method | 11  **89** | **AGREE:**  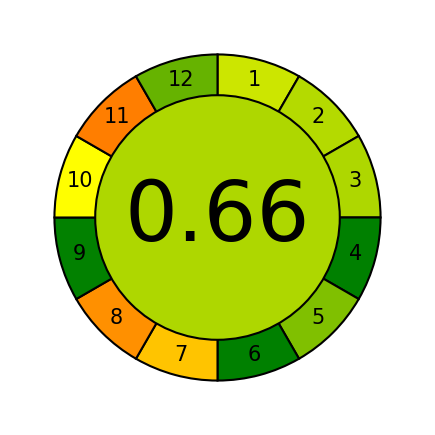 |
